# Editorial: Rising stars in radiation oncology 2022

**DOI:** 10.3389/fonc.2023.1228417

**Published:** 2023-06-21

**Authors:** TJ FitzGerald

**Affiliations:** ^1^ Department of Radiation Oncology, UMass Memorial Health Care, Worcester, MA, United States; ^2^ Department of Radiation Oncology, UMass Chan Medical School, Worcester, MA, United States

**Keywords:** radiation oncology, patient care, translational science, imaging, training, clinical trial

As patient care has matured and become increasingly complex, the fundamental skill set for the modern radiation oncologist has evolved as well. The initial generation of radiation oncologists trained in the United States and North America were taught by highly skilled mentors with expertise in surface anatomy and fluoroscopy with radiation fields designed by common understanding of the pattern of disease spread. Our first-generation mentors were critical thought leaders in applying management tools available at that time to trainees. This generation of radiation oncologists applied their expertise with the tools of the day; however, as our technology has evolved, the modern radiation oncologist requires skills commensurate with rapid technology changes. As we evolve and mature as thought leaders in the oncology practice of today, the skills required for the modern radiation oncologist both in clinical care and in basic science evolve and reach a new level of performance to match expectations of our colleagues and patients. Although it was important for radiation oncology residents to be versed in medicine and medical oncology, radiation oncology was initially a surgical subspecialty requiring clinical expertise in all surgical subspecialty disciplines. Prior to the advent of modern imaging, the physical presence of the radiation oncologist in the operating room provided invaluable information to help plan the patient with fluoroscopic imaging and detailed understanding of anatomy and pathways of tumor spread. The emergence of modern imaging and the integration of multiple imaging tools into daily operation have altered the skill set required for modern practice of radiation oncology. Today’s trainee and early graduate must be skilled to manage a multifaceted practice that requires inter- and intradepartmental interaction skills. This will often require simultaneous diverse thought processes including the ability to process multiple facts and issues in parallel for patient care and basic science research with priorities based on clinical need and availability of data. Ours is a unique discipline requiring modern thinking and a skill set commensurate to meet the challenges of modern patient care fully integrated with the rapid growth of knowledge in basic and translational science.

Medical students interested in a career in radiation oncology now require a strong academic portfolio to be recognized for admission to a radiation oncology residency. This explains in part why we attract the MD/PhD student as they can hone their academic objectives as part of the PhD component of their discipline and move their projects towards the interests of our discipline. Many will have a radiation oncologist involved in both thesis development and review. While this prepares students for the academic component of our work, the clinical side of our work requires equal attention as the modern patient has elevated expectations of our role in their care. For many patients, we are the first point of contact and the driver for follow-up care in many diverse disease sites. Therefore, our clinical talent and problem-solving skills must be outstanding. During the fourth/final year of medical school, it is helpful to rotate at a radiation oncology training site to gain recognition to faculty at competitive programs. Once choices have been submitted, it is wise to spend time in surgical subspecialty areas that play an important role in the practice of radiation oncology including gynecology, otolaryngology, neurosurgery, surgical oncology, and thoracic surgery. Each of these disciplines is integrated to oncology programs through imaging, pathology, and medical oncology; therefore, spending time in subspecialty care provides the trainee time to appreciate problems and pitfalls of each discipline and learn to interact with colleagues as members of a larger cancer care treatment community. At times, patients are overwhelmed by the number of people involved in their care and trainees learn through these interactions how to present information to patients in a manner the patient and family can understand. Discussing issues with colleagues *a priori* likewise limits the mixed messaging patients can hear from clinical care partners. In radiation oncology, we have the privilege of daily interactions with patients; therefore, often we are the first to encounter asymmetry in what was said and what was heard in patient dynamics and can work with colleagues to make certain information is provided to patients in the manner intended by the group including interpretation of imaging. Modern data and information transparency can make these interactions challenging; however, timely conversations can limit misperceptions in dialogue. Often, this is an evolving process and team members change; therefore, re-educating colleagues on these points is a continuous process. Henry Adams indicated that education is a self-renewal process, and the dialogue generated from interactions with colleagues extend into tumor boards and provide an opportunity to re-visit and re-structure care plans based on clinical status and new knowledge (1). Because we interact with every surgical and medical discipline, the modern radiation oncologist becomes both a thought leader and a bridge builder between disciplines who may be less familiar with the work scope of other departments. This becomes an opportunity for leadership within our discipline for which the modern radiation oncologist must prepare and assume responsibility. Leadership training of this nature is essential to modern practice and can be imbedded into training programs and include training for professional interactions and cultural diversity. These are important structural components to modern training and must be part of the foundation and autonomic skill set of the modern practitioner; otherwise, we risk being lateralized in this process by others who embrace and follow this approach. While our skill set significantly differs from medical and surgical colleagues, we need to find symbiosis and common ground with our colleagues to make certain patient care is applied in a seamless manner and lead conversations with oncology colleagues on the future directions of our discipline.

Modern practice requires lifelong commitment to self-improvement. In early training, neophytes to the field of radiation oncology will often focus on the abstract and discussion of a paper. As we mature in training, we learn to focus on methods/materials and the results to draw our own conclusions, which may or may not align with the talking points in the discussion of a paper. The transition in placing emphasis on data and data analysis leads the trainee to attending physician status, confident but not overly so to continue and ask colleagues and themselves salient questions about patient care and science. During this phase, specific interests mature and develop, and many radiation oncologists move forward and identify their work within a specific disease discipline. In this capacity, the radiation oncologist defines an area of clinical and academic expertise, building relationships with colleagues outside of the department. Good departments become great when colleagues with similar disease interest understand the strengths and limitations of therapy outside of their discipline. Great departments become outstanding when protocols based in translational science are developed and outcomes are published for others to review. This promotes progress and provides recognition to the group for their efforts in using science to improve patient care (2, 3). Li et al. evaluated potential imaging biomarkers based on ^18^F-AlF-NOTA-PRGD2 positron emission tomography/computed tomography and dynamic contrast-enhanced magnetic resonance imaging and response to bevacizumab, conventional concurrent radiotherapy, and temozolomide in patients with glioblastoma. Liu et al. assessed the short-term toxicity and feasibility of treating patients with early-stage breast cancer with a five-fraction stereotactic body radiation regimen for accelerated partial breast radiation.

Our field has many exceptional young talented investigators, and the portfolio for study and academic growth now extends to include a broad spectrum of topics with multiple areas of intersecting science. We have outstanding basic scientists, computer scientists, experts in physics and treatment planning, experts in big data and data management, and outstanding clinical care specialists who can apply new knowledge gained from these areas of expertise to patient care moving forward. Shang et al. analyzed treatment patterns and impact of radiotherapy for patients with unresected stage III non-small cell lung cancer in the National Cancer Institute Surveillance, Epidemiology, and End Results (SEER) database between 2001 and 2016. Through a systematic review and analysis, Wu et al. assessed the efficacy and safety of radiotherapy and radiotherapy/chemoradiotherapy and immune checkpoint inhibitors. Mo et al. assessed the SEER database from 2004 to 2015 for patients with primary lung cancer treated with radiotherapy who later developed a second primary lung cancer. Sun et al. retrospectively investigated the prognostic factors of tumor size, volume, and tumor volume reduction during concurrent chemoradiotherapy in patients with cervical cancer. Provided we remain true to our science and accurate in our reporting of data, radiation oncology will continue to grow in influence in the cancer community. Our responsibility is to continue to train talented young investigators in multiple academic endeavors to move our field forward.

## Author contributions

The author confirms being the sole contributor of this work and has approved it for publication.
